# An atypical winter outbreak of hand, foot, and mouth disease associated with human enterovirus 71, 2010

**DOI:** 10.1186/1471-2334-14-123

**Published:** 2014-03-04

**Authors:** Nan Liu, Jing Xie, Xiaoli Qiu, Leili Jia, Zhihao Wu, Yuhua Ma, Zhongqiang Wang, Peng Li, Xingbin Ren, Rongzhang Hao, Ligui Wang, Yong Wang, Shaofu Qiu, Hongbin Song

**Affiliations:** 1 Academy of Military Medical Sciences, Institute of Disease Control and Prevention, 20 Dongda Street, Fengtai District, Beijing 100071, China; 2 Department of Clinical Laboratory Sciences, Linyi People’s Hospital, Linyi 276000, China

**Keywords:** Hand, Foot, and mouth disease, HFMD, Enterovirus 71, Phylogenetic analysis, Subgenotype C4

## Abstract

**Background:**

To analyze the epidemiological characteristics and pathogenic molecular characteristics of an hand, foot, and mouth disease (HFMD) outbreak caused by enterovirus 71 in Linyi City, Shandong Province, China during November 30 to December 28, 2010.

**Methods:**

One hundred and seventy three stool specimens and 40 throat samples were collected from 173 hospitalized cases. Epidemiologic and clinical investigations, laboratory testing, and genetic analyses were performed to identify the causal pathogen of the outbreak.

**Results:**

Among the 173 cases reported in December 2010, the male–female ratio was 1.88: 1; 23 cases (13.3%) were severe. The majority of patients were children aged < 5 years (95.4%). Some patients developed respiratory symptoms including runny nose (38.2%), cough (20.2%), and sore throat (14.5%). One hundred and thirty eight EV71 positive cases were identified based on real time reverse-transcription PCR detection and 107 isolates were sequenced with the VP1 region. Phylogenetic analysis of full-length VP1 sequences of 107 Linyi EV71 isolates showed that they belonged to the C4a cluster of the C4 subgenotype and were divided into 3 lineages (Lineage I, II and III). The two amino acid substitutions (Gly and Gln for Glu) at position 145 within the VP1 region are more likely to appear in EV71 isolates from severe cases (52.2%) than those recovered from mild cases (8.3%).

**Conclusion:**

This outbreak of HMFD was caused by EV71 in an atypical winter. EV71 strains associated with this outbreak represented three separate chains of transmission. Substitution at amino acid position 145 of the VP1 region of EV71 might be an important virulence marker for severe cases. These findings suggest that continued surveillance for EV71 variants has the potential to greatly impact HFMD prevention and control.

## Background

Hand, foot, and mouth disease (HFMD), primarily a disease of young children, is caused by a virus belonging to the group enteroviruses. EV71 is known to be a causative agent of HFMD, herpangina, aseptic meningitis, paralysis, and meningoencephalitis [1]. HFMD usually presents with symptoms including fever and a characteristic rash associated with the limbs, mouth and skin. In severe disease such as caused by EV71, however, the patients may develop nervous system diseases such as aseptic meningitis, encephalitis, brainstem encephalitis, encephalomyelitis, and poliomyelitis-like syndrome as well as neurogenic pulmonary edema and myocarditis, resulting in high morbidity and mortality [2].

EV71 was first isolated in the United States (California) in 1969 and by the mid-1970s, EV71 outbreaks, characterized by central nervous system complications occurred in succession in Bulgaria and Hungary [3]. In the late 1990s, EV71 emerged in East Asia. In 1997, 2628 cases in Malaysia were reported, with 34 deaths [4]. One year later, an estimated 1.5 million cases occurred in Taiwan; 405 cases developed severe neuropathic complications with 78 deaths [5]. In 1999, an EV71 pandemic occurred in Perth, Australia; 6000 cases were reported in 6 months; 29 cases developed severe disease [6]. Subsequently, numerous outbreaks have been documented in eastern and southeastern Asia, including Singapore [7], South Korea [8], Malaysia [9], Japan [10], Vietnam [11] and mainland China. In 2008, a large scale outbreak of HFMD occurred in Anhui Province, China; 3321 cases were reported with 22 deaths [12].

HFMD outbreaks associated with EV71 exhibit a significant seasonal pattern with a peak in summer and low incidence in winter [13,14], however the number of HFMD cases increase significantly with increasing temperature and relative humidity [13,15]. In this study, we describe an atypical winter outbreak of HFMD from November 30 to December 28, 2010, in Linyi City, Shandong Province, China, during which 173 cases were admitted to the Linyi People's Hospital. In this study, we analyzed the epidemiological characteristics of this outbreak and the molecular epidemiology of EV71, with an attempt to provide scientific evidence for the prevention and control of HFMD.

## Methods

### Case information and clinical samples

One hundred and seventy three patients admitted to Linyi City People's Hospital were enrolled in the study. Case information was obtained from the hospital information system including age, gender, onset date, place of onset and clinical information. All 173 cases were diagnosed according to a standardized clinical case definition of HMFD [16]. HFMD was defined as fever, accompanied by herpangina and rashes on the hands and feet, with or without buttock involvement. Severe HFMD patients presented with obvious symptoms of nervous system involvement and severe complication, such as myoclonus, encephalitis, acute flaccid paralysis, pulmonary edema, or heart failure. To confirm the diagnosis of HMFD, 213 specimens (173 stool and 40 throat swabs) were collected for enterovirus detection and molecular typing. Throat swabs were immediately immersed into sterile tubes containing viral transport medium (VTM). The study protocol was approved by the Medical Ethics Committee of Academy of Military Medical Sciences.

### Virus isolation

Approximately 0.5 g of a fecal sample was put into a 1.5 ml centrifuge tube with a glass bead. After addition of 1 ml of phosphate buffer, the solution was vortexed for 1 min and centrifuged at 4000 rpm for 10 min. Fecal supernatants and the VTM from tubes containing throat samples were filtered through a 0.22 or 0.45 μm syringe filter (Pall, Ann Arbor, MI, USA). Filtrates were cultured with human rhabdomyosarcoma cells (RD cells) at 37°C and 5% CO_2_ for 7 days. Cells were observed daily for cytopathic effects (CPE). Each specimen was passaged blindly at least 3 times. Cells demonstrating an observed CPE were repeatedly frozen and thawed 3 times to allow for RNA extraction and identification using molecular biology approaches, and then cell cultures and RNAs were stored at −80°C. If no specific CPE was observed cell culture was interpreted as negative.

### Nucleic acid extraction and molecular typing

Viral RNA was extracted from clinical specimens and viral cultures using a QIAamp Viral RNA Mini Kit (Qiagen, Germany) according to the manufacturer's instructions. All RNA samples were examined by real time reverse-transcription PCR (rt RT-PCR) using a set of Pan-EV (EV universal primer) probe and primers; positive samples were tested by rt RT-PCR for EV71 and CA16 using specific primers and probes [17]. Rt RT-PCR was performed using AgPath-ID™ One-Step RT-PCR reagents (Applied Biosystems, Foster, CA, USA). Total DNA was extracted from 40 throat specimens using QIAamp DNA Blood Mini Kit (Qiagen, Germany). Commercial available multiplex PCR assays (Seeplex® RV12 ACE, Korea) was used for testing respiratory viruses of 40 throat specimens, including human adenovirus, coronaviruses, parainfluenza viruses, influenza A virus, influenza B virus, respiratory syncytial virus A and B, and bocavirus. Amplification was performed using an IQ™5 quantitative real-time PCR system (BioRad, Hercules, CA, USA).

Full-length VP1 sequences from EV71 isolates were amplified with the primers EV71-VP1-F, GGKGCRCCCAAYACWGCYT and EV71-VP1-R, CCVCCRCAATCHCCWGGYT, resulting in a 981-bp amplicon. PCR was performed using a GeneAmp 9700 Thermal Cycler (Applied Biosystems). PCR products were detected by agarose gel electrophoresis, and products were purified and sequenced with an ABI PRISM 3730 Genetic Analyzer (Applied Biosystems). An online Blast Search Tool (http://blast.ncbi.nlm.nih.gov/Blast.cgi) was used to compare the sequences and determine the types. The constituent ratio of amino acid variation between severe cases and mild cases strains was compared with Chi-square test by SAS software (version 9.0; SAS Institute).

### Phylogenetic analysis

Multiple sequence alignments were performed by using the MEGA software version 5.2 and the Clustal W program to determine nucleotide and amino acid sequence similarities. Phylogenetic trees were constructed in MEGA using the neighbor-joining (NJ) cluster algorithm with evolutionary distances estimated using the Kimura 2-parameter model; bootstrapping was performed using 1,000 pseudo-replicates [18].

### Nucleotide sequence accession numbers

The full sequences of VP1 from this study are deposited in GenBank with the following accession numbers: KF853482-KF853549, KJ185132-KJ185170.

## Results

### Epidemiological analysis and clinical symptoms

**Figure 1:**
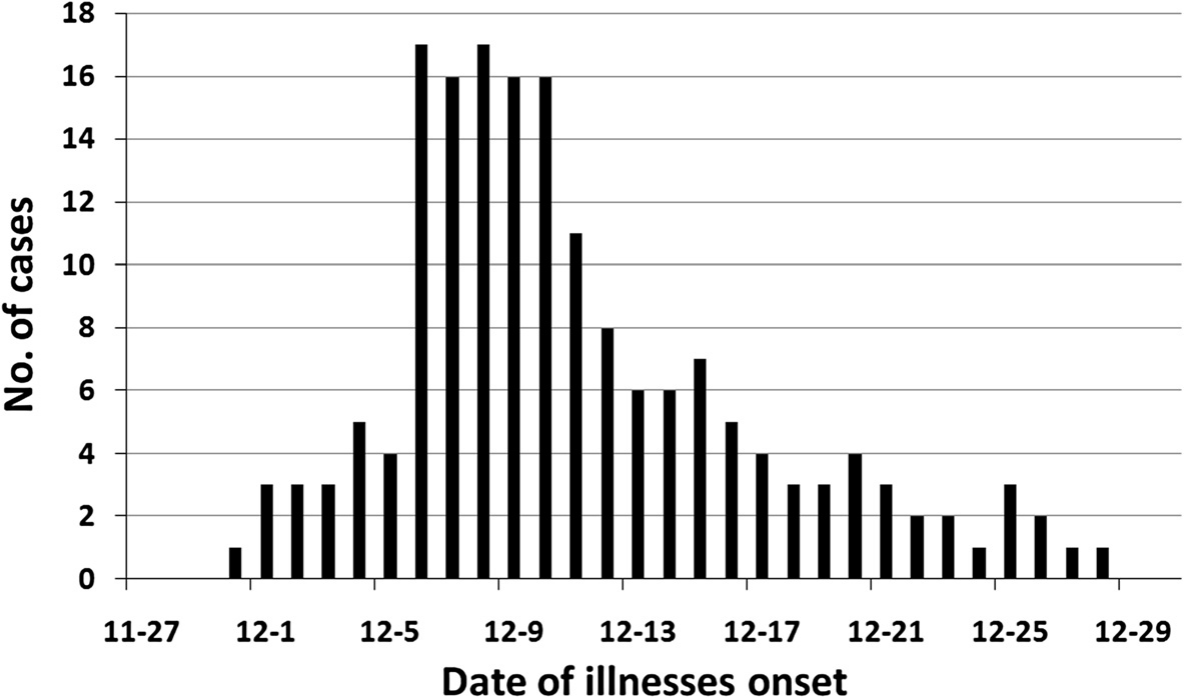
Epidemic curve of the HMFD outbreak in Linyi City, Shandong Province, China, 2010.

A total of 173 HFMD cases (including 23 with severe presentation) were diagnosed and treated in Linyi People's Hospital from November 30 to December 28, 2010 (Figure 1). The majority of patients were children aged < 5 years (95.4%) with the male–female ratio of 1.88:1. Cases presented from 9 counties or districts; although the incidence was greatest in Lanshan District (35.8%) and Fei County (31.2%).

All cases had fever and the characteristic rashes associated with the limbs, mouth and skin. Severe cases were associated with meningitis (n = 15) or more severe neurological complications (meningoencephalitis, n = 3; neurogenic pulmonary edema, n = 5); no deaths were observed. Although some patients developed respiratory symptoms including runny nose (38.2%), cough (20.2%), and sore throat (14.5%), testing with multiplex PCR assays of a subset of throat samples (n = 40) for respiratory viruses including human adenovirus, coronaviruses, parainfluenza viruses, influenza A virus, influenza B virus, respiratory syncytial virus A and B, and bocavirus were all negative. A small number of patients also reported nausea, or experienced vomiting, diarrhea, and other gastrointestinal symptoms. Most cases were accompanied by characteristic neurological symptoms including drowsiness and convulsions (Table 1).

**Table 1:** Summary of clinical characteristics associated with an outbreak of HMFD due to EV71 in Linyi City, Shandong Province, China in 2010

**Characteristics**	**Value (n = 173)**
Male	113 (65.3)
Age group	
<1	8 (4.6)
1	50 (28.9)
2	52 (30.1)
3	43 (24.9)
4	12 (6.9)
5	5 (2.9)
>5	3 (1.7)
Clinical symptom	
Fever	173 (100)
Rash	173 (100)
Runny nose	66 (38.2)
Cough	35 (20.2)
Pharyngalgia	25 (14.5)
Nausea	13 (7.5)
Vomiting	7 (4.0)
Diarrhea	5 (2.9)
Headache	5 (2.9)
Lethargy	65 (37.6)
Convulsion	34 (19.7)
Meningitis	15 (8.7)
Meningoencephalitis	3 (1.7)
Neurogenic pulmonary edema	5 (2.9)
Severe cases	23 (13.3)
Mild cases	140 (86.7)

### Laboratory identification

All RNA samples were examined by rt RT-PCR for the presence of any detectable enterovirus (Pan EV screen) and then specifically for EV71 and CA16. Among the 213 specimens, an amplicon was evident in 148 of the samples, taken from 138 cases, using the Pan-EV primers and probes. Using primers and probes specific for EV71, the 148 Pan EV-positive specimens were also positive; stool specimens were positive from 138 cases, and 10 cases who had a throat swab and fecal sample both tested positive. Primers and probes specific for CA16 were negative in all the samples. Viral samples from the ten patients with two positive samples were indistinguishable. In total, 107/213 (50.2%) samples from 107 individual cases were culture-positive for EV71; identification was confirmed by rt RT-PCR amplification and sequencing of VP1 regions; 23 isolates (10.8%) were recovered from severe cases and 84 isolates (39.4%) from mild cases.

### EV71 phylogenetic analysis

**Figure 2:**
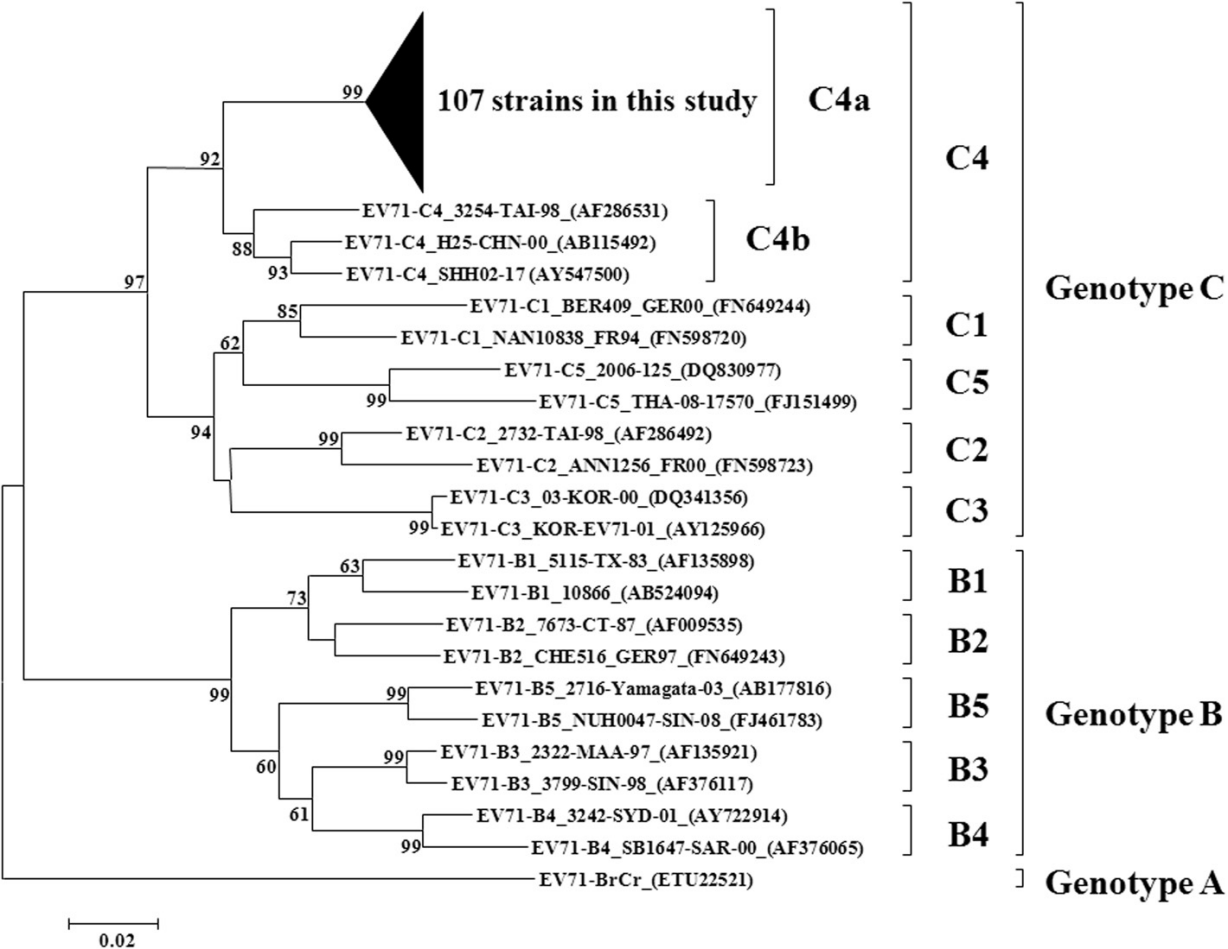
Dendrogram showing the phylogenetic relationships of 107 EV71 isolates in this outbreak and other genotypes of EV71 isolates from GenBank based on VP1 sequence alignment, which was constructed using Mega software (version 5.2).

**Figure 3:**
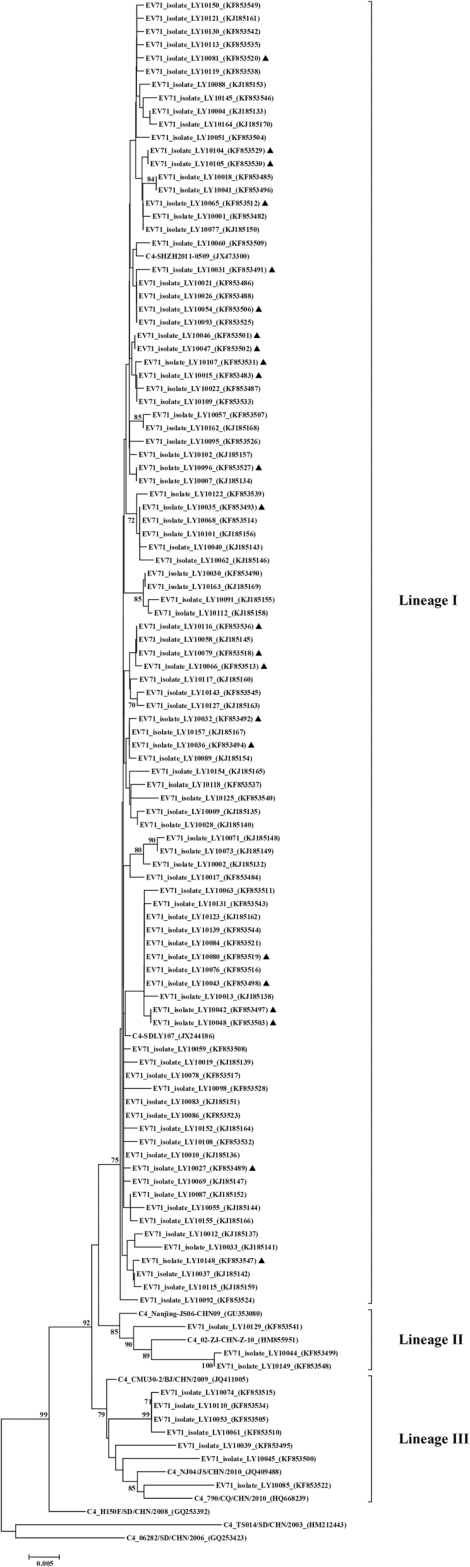
**Phylogenetic tree of 107 EV71 isolates in this outbreak and related C4a subgenotype strains.** Accession numbers are given in parentheses. ▲, isolates from severe cases. LY, isolates from this study; SD, Shandong Province; ZJ, Zhejiang Province; BJ, Beijing City; JS, Jiangsu Province; CQ, Chongqing City.

In order to analyze molecular epidemiological characteristics of the EV71 isolates in this study, 107 EV71 isolates were selected from this outbreak, and VP1 sequences were compared to those from 10 EV71 isolates circulating in China and 19 additional EV71 strains (A, B1- B5, and C1 - C5) isolated from children living in other countries (Figure 2). Sequence alignments showed the 107 Linyi EV71 VP1 sequences shared 96.2-100% nucleotide identity. Sequences of the VP1 region of the Linyi EV71 isolates were closely related to the predominant EV71 isolates from China, and all isolates belonged to subgenotype C4. Subgenotype C4 can be divided into two groups (C4a and C4b) [19]. According to the phylogenetic analysis (Figures 2 and 3), all of the Linyi EV71 isolates belong to C4a; isolates were further divided into three lineages (lineage I, II, and III) with mean intra-lineage p-distances of 0.007, 0.017 and 0.016, respectively. The majority of the outbreak isolates (97/107) belonged to lineage I which contained all of the isolates from the severe cases; these isolates were mainly found in three districts or counties of Linyi City, Shandong Province (Lanshan District, Fei County and Hedong District). Lineage II and lineage III had three and seven outbreak isolates, respectively. VP1 nucleic acid dissimilarity among the three subgroups ranged from 2.2%- 3.3%; these results suggest that the outbreak likely consisted of three main transmission chains, with lineage I being the predominant contributor.

### Amino acid sequence analysis

**Figure 4:**
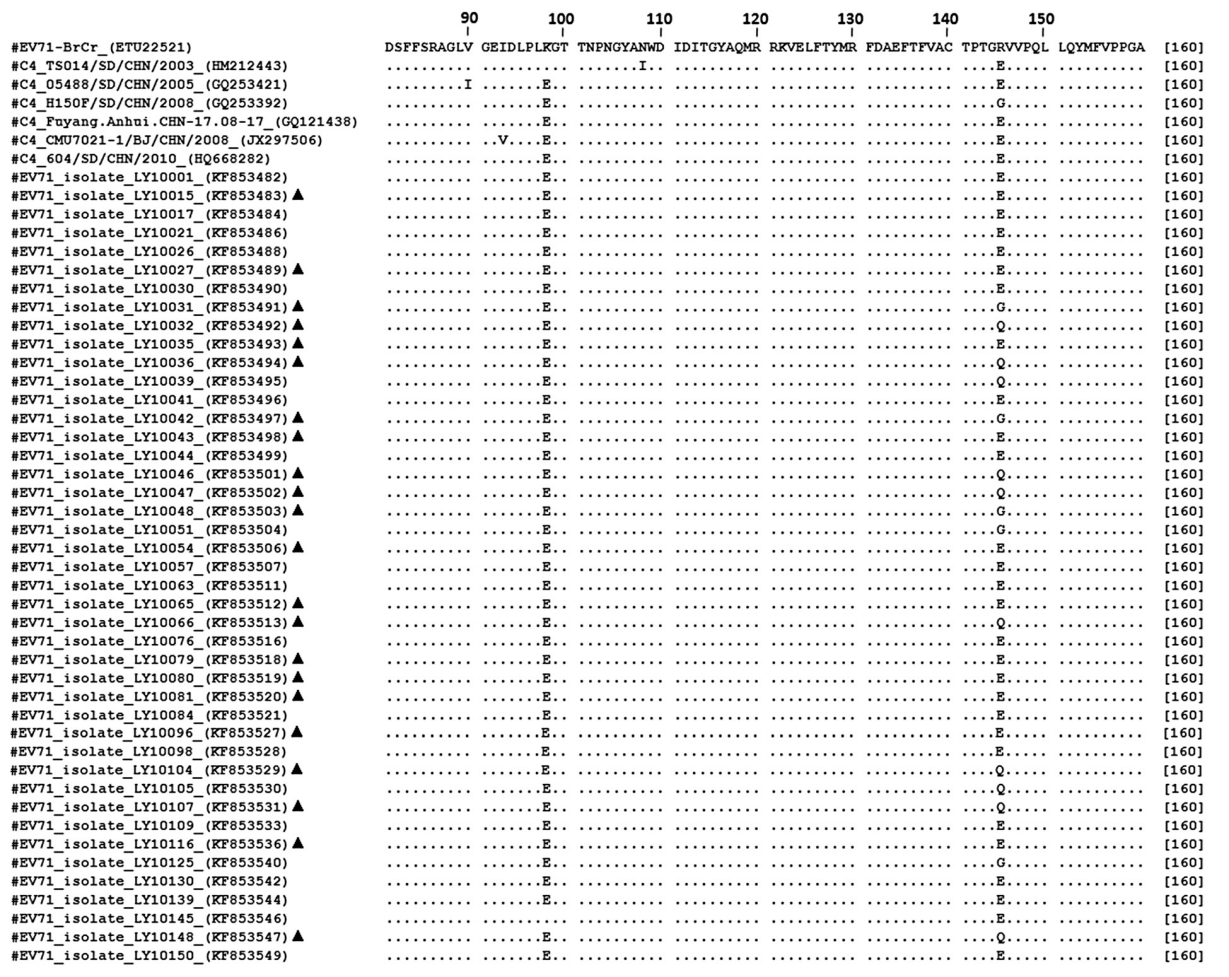
**Amino acid sequence alignment of the full length VP1 region of the EV71 virus isolates in Linyi City, Shandong Province, 2010.** Amino acid sequences were numbered according to the sequence of EV71_BrCr (Genbank accession number: ETU22521). Identical residues are indicated as dots. Amino acids that differ from the consensus sequence are shaded. ▲, isolates of severe cases.

Comparison of the translated VP1 amino acid sequences (297 aa) of the EV71 isolates used in this study indicated that the isolates were 98.6% identical to one another; variable amino acid residues were at positions 31, 43, 98, 145, 282, 262 and 293. Substitution at amino acid position 145 of VP1 appeared to be significantly different between severe cases and mild cases strains. Two amino acids, Gly and Gln, were significantly more likely to appear in the VP1 of EV71 recovered from severe cases (12/23, 52.2%) than in mild cases (7/84, 8.3%; P < 0.001) (Figure 4).

## Discussion

HFMD epidemics in China mainly occur in the spring and summer [20-22], when hot and humid weather is conducive to the propagation and spread of the virus [23]. However, the Linyi 2010 HFMD outbreak occurred in December, which is relatively uncommon. According to meteorological parameters provided by the Meteorological Bureau of Shandong Province, the mean temperature of Linyi City during the winter of 2010 was 0.6°C higher than that in 2009. Flett et al. suggested that atypical seasonality of HFMD outbreaks during winter might be related to unusually mild temperatures [24]. Research in Hong Kong has also indicated that altered disease etiology of HFMD might be explained by increased winter temperatures [25]. It is reasonable to speculate that the short-term changes in weather variables may affect the seasonality of HFMD. However, cold weather increases the probability that populations will gather in confined spaces such as houses and indoor play areas; increasing the likelihood of unwanted exposures. In addition, more upper respiratory tract infections occur during the winter, resulting in a decrease in immune functions among pediatric patients, which might contribute to increasing incidence of HFMD.

The epidemiology of EV71 in China since this virus was first detected in 1998 has shown that only subgenotype C4 is in endemic circulation [19]. Phylogenetic analysis suggests that three EV71 variants contributed to the 2010 Linyi outbreak and share strong similarities to some EV71 viruses circulating previously in Shandong Province. However, no obvious evidence revealed the transmission relationship between strains from this outbreak and other provinces in China. Therefore, we speculate that this uncommon winter outbreak did not occur in isolation, but was related with local epidemic occurring in the region during the spring and summer of 2010. It is probable that the change of climatic parameters contributed to the occurrence of this atypical seasonal outbreak but the extent of this contribution needs to be further examined.

Subgenotype C4a is geographically broadly distributed and genetically variable; it is estimated to undergo 4.97 × 10^−3^ substitutions per nucleotide per year, a rapid rate of change [26]. Phylogenetic analysis shows that subgenotype C4 should be re-designated as a novel genotype, D [27,28]. Thus, the three lineages may represent different EV71 transmission modes in this region; this hypothesis should be clarified by further molecular epidemiological studies.

Amino sequence analysis showed one primary variation between viruses isolated from severe and mild cases of HFMD, substitution of the amino acid residue at position 145. Several studies have revealed that this change in the VP1 region of EV71 may play an important role in virulence. Residue 145 is reported to be located in the DE loop which contains neutralizing antigenic sites; this loop is located in the canyon rim, a region involved in the receptor binding for enterovirus and rhinovirus [29-32]. Selection pressure analysis of many VP1 sequences of EV71 showed that amino acid 145 was a positive selection site [33,34]. Mutation of glutamine to glutamic acid on VP1 145 region cooperatively promote viral binding and RNA accumulation of EV71, contributing to viral infectivity *in vitro* and mouse lethality *in vivo*[35]. Substitution of glycine to glutamic acid at the same position might increase the efficiency of uncoating upon specific binding of the virion to the receptor molecule on the target cells in NOD/SCID mice and subsequently facilitate the infection of a mouse adapted EV71 strain [36]. Although it has been demonstrated that the amino acid substitution of Gly to Glu at position 145 of VP1 could increase EV71 virulence in mice, Glu to Gly/Gln/Arg substitutions may enhance virulence in humans [37]. Consistent with this result, isolates from severe cases in our investigation were significantly more likely to carry the Gly/Gln at amino acid 145 than those from mild cases. This substitution may influence the virus binding to antibody or receptor, thereby affecting the virulence of the virus [37].

## Conclusions

In conclusion, HFMD caused by EV71 has become a global concern in recent years. In February 2008, HFMD was officially categorized as a Class C infectious disease in China. Further studies on the transmission patterns of EV71 using approaches in molecular biology are therefore warranted, and recognizing EV71 isolates with increased virulence should be a priority for HFMD prevention and control efforts. Moreover, monitoring the genetic variation of EV71 may be useful in facilitating the development of effective EV71 vaccine candidates on Chinese market and evaluating their effects of prevention and control of the EV71 infections.

## Competing interests

The authors declare that they have no competing interests.

## Authors’ contributions

NL performed the experiments, and drafted the manuscript. XLQ, YHM and XBR participated in the collection of specimens and clinical data. ZHW and ZQW assisted with clinical data processing. LLJ, PL and JX carried out the molecular genetic studies, and participated in the sequence alignment. JX participated in the revision of the manuscript. LGW performed the statistical analysis. RZH and YW conducted the literature review. HBS and SFQ designed and conducted this study, and revised this manuscript. All authors read and approved the final manuscript.

## Pre-publication history

The pre-publication history for this paper can be accessed here:

http://www.biomedcentral.com/1471-2334/14/123/prepub
